# Paediatric Long Bone Fractures Managed with Elastic Intramedullary Nails: A Retrospective Study of 30 Patients

**DOI:** 10.7759/cureus.7847

**Published:** 2020-04-27

**Authors:** Susanta Khuntia, Shakti Swaroop, Bishnu P Patro, Subhrajyoti Sahu

**Affiliations:** 1 Orthopaedics, All India Institute of Medical Sciences, Bhubaneswar, IND; 2 Microbiology, Maharaja Krishna Chandra Gajapati (MKCG) Medical College, Berhampur, IND

**Keywords:** intramedullary nail, elastic, pediatric fractures

## Abstract

Elastic intramedullary nailing or titanium elastic nail (TEN) is an alternative method for the treatment of various pediatric long bone fractures. Titanium nails are preferred over plaster cast and stainless steel nails for children older than six years. Our series included 30 paediatric long bone fractures who were managed with TEN. The mean age was 9.3 years and the mean follow-up period was 28 months. A mean time of 10 weeks was recorded for the union of all fractures. The occurrence of superficial infection in three cases healed with antibiotics and minor debridement. Limb length discrepancy was seen in three cases of femur fracture, which was functionally insignificant, but it may be a potential problem needing close follow-up until skeletal maturity is attained. An elastic intramedullary nail or TEN in long bone fractures in children is a safe and minimally invasive technique that achieves stable reduction, especially in long spiral fractures till union. We attained successful union and good results in all our 30 cases with long bone fractures in children. A few complications of hardware prominence were resolved with implant removal. Long-term studies with a comparison to casting techniques in paediatric long bone fractures are required.

## Introduction

The constantly evolving methods of treatment of fractures in long bones in children have shown a preference for the use of intramedullary elastic nails. The majority of the long bone fractures in the skeletally immature is being treated by conservative methods. Conservative treatment remains the mainstay for long bone fractures in children below the age group of six years as the remodelling ability of the immature bone in children is excellent [1]. However, the management of long bone fractures for ages between six years and 16 years is controversial [2]. Plaster casting for the fracture of long bones in children less than six years and intramedullary nailing in adolescent above 16 years was suggested by Metazziau. Elastic intramedullary nailing was preferred in older children due to poor tolerance of immobilization and an uncomfortable cast. In addition, there is less potential for the correction of mal-alignment in long bone fractures in older children nearing adolescence. Unstable fractures, i.e., where close manipulation fails to maintain the reduction with a release of the reducing force, with shortening more than 10 mm or angular and rotational deformity more than 15 degrees, required surgical options to maintain reduction and preserve function [3]. Various methods of fixation are available for the stabilization of long bone fractures in children, however, the introduction of flexible elastic nails has changed the management for shaft-of-femur fractures in children significantly [4-5]. Many other intramedullary nails, such as Rush nails and Ender’s nails, are available for the treatment of long bone fractures in children, but they lack rotational stability. As we know, the management of femur fractures in the paediatric age group of up to six years is mostly conservative, but we used a titanium elastic nail (TEN) in the management of all long bone fractures in children more than the age of six years and less than 15 years and share our experience to add to the evidence in the management of long bone fractures in the paediatric age group [5-6].

## Materials and methods

We performed a retrospective study of paediatric long bone fractures operated with an elastic intramedullary nail. Cases were included from January 2014 - February 2016. There were 21 male and nine female patients included in the study. The mean age at the time of surgery was 10 years (six to 15 years). There were 15 femoral fractures, six tibia fractures, eight forearm fractures, and one humerus fracture. Modes of injury were self fall, road traffic accidents, fall from a height, and two cases of abuse. All injuries were closed except one tibia, which was compound grade II with de-gloving of the skin and soft tissue. The decision for surgery was based on anatomical reduction and fracture instability.

Intramedullary nailing was done under anaesthesia and possible aseptic precautions. Under image intensifier, the entry point was made with an awl; the fracture was reduced with the help of an F-tool. Once planned for surgery, nails were passed up to the fracture end and after achieving acceptable reduction, nails were further introduced beyond the fracture site. 

We measured the narrowest diameter of the medullary canal with a ruler. The nail diameter chosen was no more than 40% of the width of the canal or one-third of the narrowest part of the intramedullary canal. Two nails with the same diameter were selected to balance the opposing bending forces and avoid mal-alignment.

Both nails were contoured into a bow shape, keeping in mind that the apex of the bow will lie over the fracture, with the tip pointing to the concave side of the bowed nail. The bow in each nail was kept similar for a balanced effect to counteract malaligning forces.

In the femur, humerus, and ulna, a retrograde insertion of the nail was done, and in the radius and tibia, antegrade insertion of the nail was preferred. After crossing the fracture, the nails were passed up to the metaphyseal zone, with each tip facing away from each other to give three-point fixation in the metaphyseal bone. The nails were cut within 2 cm long and bent along the cortex of the bone to prevent any soft tissue impingement.

Intravenous injection ceftriaxone was given preoperatively and three doses were administered postoperatively over a period of two days. In six children, open reduction was required to achieve passage of the nail. In the case of a tibia and fibula fracture at the middle 1/3rd shaft with a grazed laceration over the medial malleolus, secondary skin grafting was done after the development of granulation tissue. In four cases of femur fractures and three tibial fractures, additional casts were applied for additional rotational stability due to the long spiral nature of the fracture. Ambulation was allowed after four weeks upon removal of the POP cast and the majority of the children tolerated weight-bearing well at the end of four weeks. For patients who were not put on postoperative casts, immediate active-assisted hip, knee, and ankle movement were started from the next day of surgery.

## Results

All 30 fractures went on to unite. We have evaluated the time to union and complications along with the limb length discrepancy, pain and rotational malalignment to measure the outcome according to the FLYNN criteria outcome scoring system [7]. The mean time to union was 10 weeks (mean - 9.93 weeks). It ranged from six to 16 weeks as per the fracture type and long bone involved. The mean time for union of the femur was 10 weeks and for the tibia, it was 11 weeks. Upper limb fractures took less time to unite at a mean of eight weeks.

The mean follow-up period was 26 months (18 - 44 months). In most cases, the fracture was manipulated by closed methods and the nail introduced across the fracture site but in six cases, we faced difficulty and an incision was made to reduce the fracture site directly for passage of the nail. It did not result in any delay in fracture union or infection. Rotational malalignment was not present, which was examined clinically intraoperatively and after fracture union.

**Figure 1:**
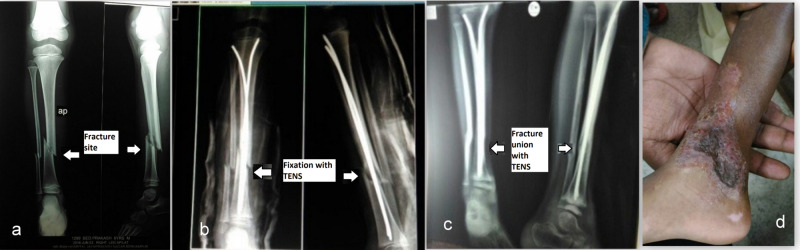
Tibia and fibula fracture managed with TEN a. Fracture of both bone legs preoperative radiograph, b. Immediate postoperative radiograph showing TEN, c. Seven months postoperative radiograph showing a united fracture, d. Clinical photo showing healed wound over the medial aspect ankle TEN: titanium elastic nail

**Figure 2:**
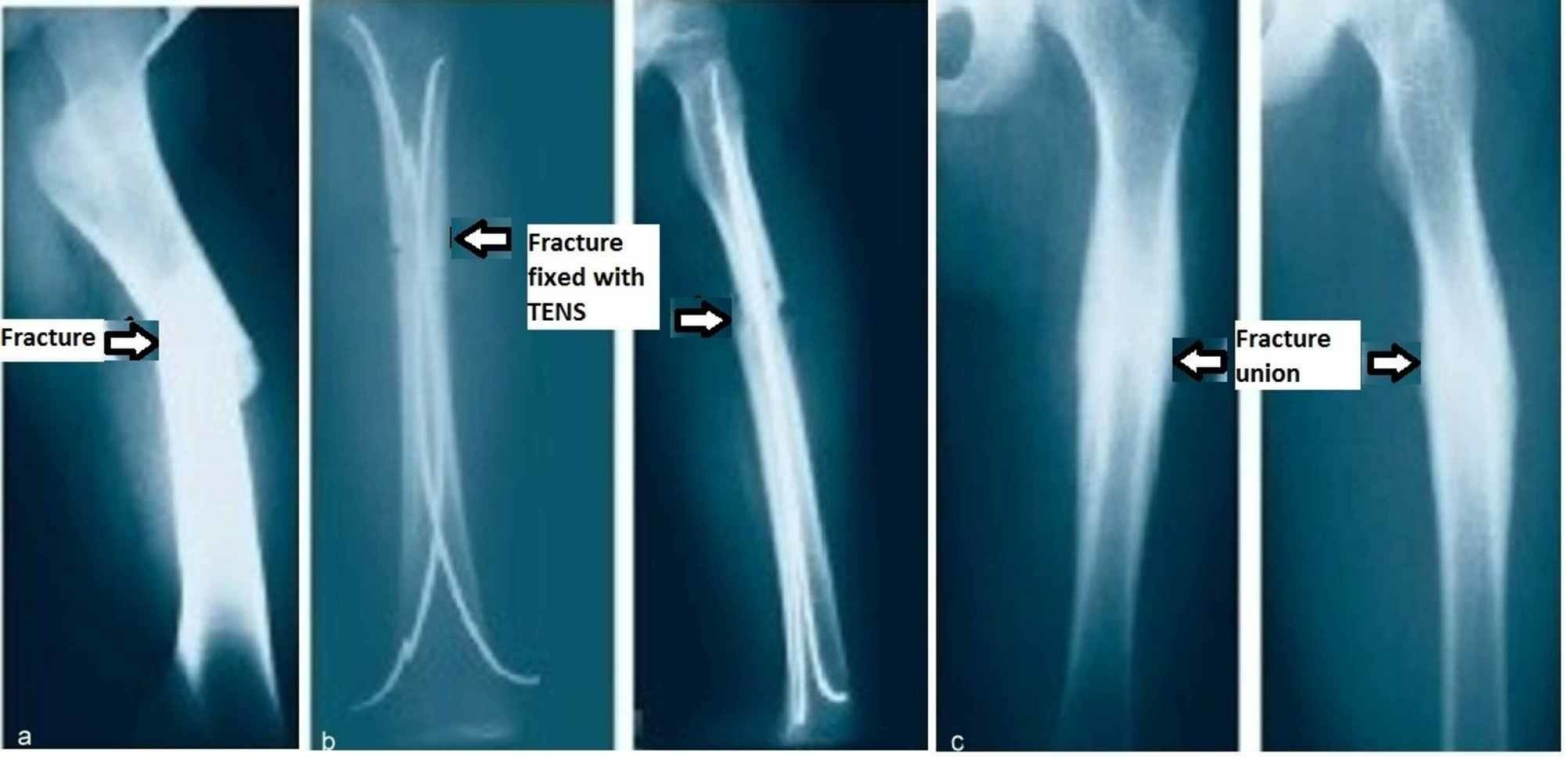
Femur shaft fracture managed with TEN a. Femur shaft fracture preoperative radiograph, b. Immediate postoperative radiograph with TEN, c. Six-month postoperative radiograph showing fracture union and implant removed TEN: titanium elastic nail

**Figure 3:**
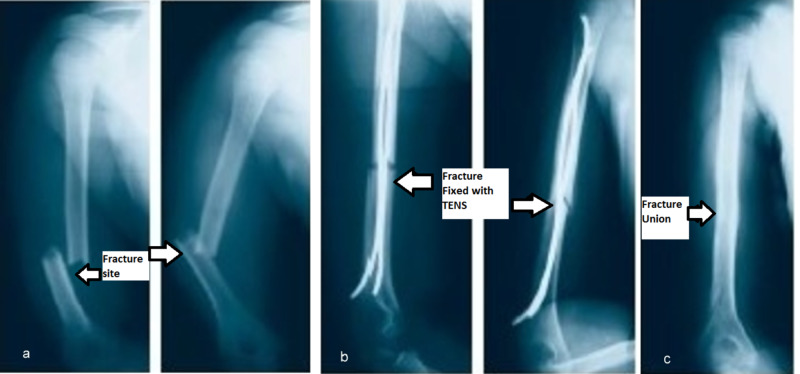
Displaced fracture shaft humerus managed with TEN a. Humerus fracture preoperative radiograph, b. Immediate postoperative radiograph showing fracture fixation with TEN, c. Radiograph showing fracture union achieved and implant removal at six months TEN: titanium elastic nail

**Figure 4:**
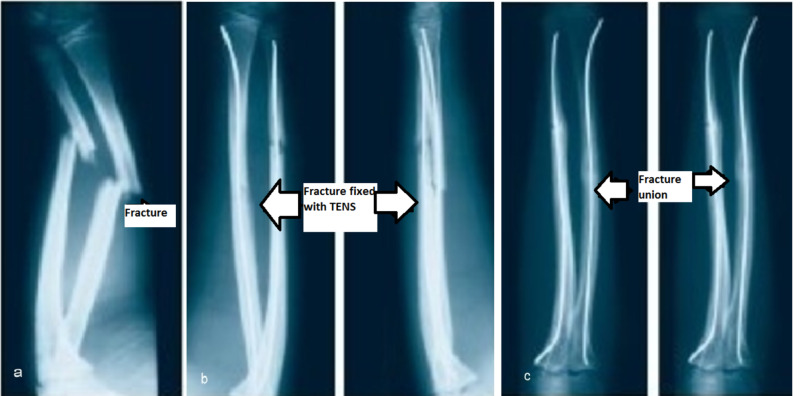
Both bone fracture forearm fracture managed with TEN a. Both bone forearm fracture preoperative radiograph, b. Immediate postoperative radiograph with TEN in situ, c. Six months postoperative radiograph with fracture union TEN: titanium elastic nail

Complications were few. Three cases had a superficial wound infection: two tibias and one femur. They all resolved with minor debridement and IV antibiotics. Four cases had nail end prominence, three femurs and one radius. The implant was removed in all cases after the fracture united. In two femur fractures, the entry point was a bit anterior and caused the nail to protrude into the knee joint, causing pain and effusion. They resolved successfully on the removal of the nail. See Figures 1-4.

Satisfactory function was achieved in all 30 children as per Flynn criteria [7]. Limb length discrepancy of more than 1 cm, i.e., the operated limb longer than the normal limb was seen in three femur fractures, but it was not functionally significant. However, this needs further follow-up until skeletal maturity to look for compensation of limb length discrepancy.

## Discussion

Considering the increase in pediatric long bone fractures in recent times, it is important to reduce the morbidity of these children and reduce the burden of prolonged immobilization. In children nearing the adolescent age group (>12 years), it is difficult to maintain the reduction in the cast. They are less tolerant of a plaster cast or traction and the residual deformity has less potential to remodel. Delayed rehabilitation due to a slower healing process as compared to younger children is also a drawback of conservative management in this age group. Femur fractures in the age group of six to 16 years have been recommended to be fixed with elastic intramedullary nails and have a good outcome with early rehabilitation [2,7].

There was a limited initial experience with the use of Kuntsher nails and Rush nails in children. Insertion through the metaphyses of skeletally immature bones was difficult due to the rigidity of these nails [8]. Ender's nails were designed to solve this but multiple Ender's nails were required most of the time and stacked into the medullary canal to achieve fracture stability [9-10]. But, as stated by Ligier et al., Ender's nail is not elastic and flexible enough for pediatric fractures [10]. The titanium flexible nail has an advantage over the Ender’s nail due to its design and material. The modulus of elasticity of 316L stainless steel is 187 GPa, making it 80% stiffer than the titanium (105 GPa) alloy. Titanium’s elasticity limits the extent of permanent deformation during insertion [7].

Even 2 mm nails have adequate stiffness and elasticity as compared to a Kirschner wire or stainless steel pin of the same diameter [7]. The advantages of TEN in pediatric fractures are that these nails are affordable; their modulus of elasticity, they do not disturb the blood supply of the femoral epiphysis; with proper bending, they provide a three-point fixation; no plaster is required; and removal of the nails can be done very easily [11]. Also, the micromotion allowed by the elasticity of the fixation promotes an external bridging callus. Being a closed procedure, the periosteum and fracture hematoma remain intact, thereby reducing chances of infection.

Other methods of surgical fixation are plate osteosynthesis, external fixation, and interlocking intramedullary nails. Plate osteosynthesis is a popular choice due to its ability to achieve excellent reduction. Its disadvantages are large tissue dissection, longer duration of immobilization, risk of delayed union or non-union due to loss of fracture hematoma, infection, and the need for plate removal [12]. The external fixators do provide good stability and early mobilization, but the risk of pin tract infections and longer time for weight-bearing are its shortcomings [13]. Avascular necrosis of femoral head, coxa valga have been reported with interlocking nails when used in skeletally immature patients [14-15]. Buford et al. have reported good results with intramedullary interlocking femoral nails in the age group of 11-16 years, avoiding the pyriformis fossa as entry site [16]. Titanium elastic nail has an advantage over other methods of fracture stabilization because it is a simple load-sharing internal splint that doesn't violate open physis and allows early mobilization while maintaining alignment [2].

In children who are heavy or very muscular, it is difficult to maintain the reduction in the plaster cast. Again in children with neuromuscular conditions or bone fragility, immobilization is to be avoided. TEN remains the first choice when compared to plating because it avoids the zone of pressure at the interface between the rigid plate and the fragile cortical bone. The nails function as a scaffold and prevent deformity of the bone. In multiple fractures, polytrauma, head injuries, and other conditions that necessitate intensive nursing care, TEN should be preferred over cast immobilisation [6].

Fracture geometry and location are also important when deciding to use elastic nails. Transverse, short oblique, and minimally comminuted fractures are suitable for titanium elastic nail as stated by Flynn et al. [17]. Metaizeau has also suggested that if one is ready to face certain levels of difficulty while operating and is experienced enough, then comminuted, long spiral, or long oblique fractures can also be managed with elastic nails [6]. Narayanan et al. recommended that transverse, short oblique, and short spiral fractures with minimum comminution within five to 12 years were the best indications of TEN [18]. Lascombes et al. suggested that TEN could be recommended in diaphyseal femoral fractures of children beyond the age of six and till they attained fusion of the physis [19]. TEN does not provide adequate stability in comminuted, long oblique, or spiral fractures. Adequate immobilization postoperatively is required if such fractures are fixed with TEN [2].

Apart from femur shaft fractures in children and adolescents, the titanium elastic intramedullary nail has been recommended in other long bone fracture management as well. Vrsansky et al. treated 308 pediatric fractures with the flexible nailing technique and achieved union and satisfactory function in all children. However, they also mentioned that these elastic intramedullary nails not to be used in children below five years [20].

In shaft fractures of the upper limbs, surgical procedures are increasing to prevent functional deformity of the forearm and cosmetic deformity of the humerus [21]. Sahu et al. have reported 35 out of 40 patients with excellent results in paediatric both bone forearm fractures managed with TEN [22]. Good results have also been reported by Kapila et al. in pediatric both bone fractures treated with the titanium elastic nailing system (TENS) [23].

Humerus fractures have also been recommended by several studies to be managed by TENS, when indicated with better results and functional outcome than casting [24-25].

Tibia fractures in the paediatric age group also show promising results with TENS, especially in cases with unstable reduction, soft tissue compromise, or polytrauma. Closed reduction and casting, however, still remains the mainstay of pediatric tibia and humerus fractures [26-27]. Allen et al. in his study compared the outcome of plating versus TENs for femur fracture in children between five to 12 years and reported that plates resulted in a longer operative time, increased costs, and equivalent pain compared with TENs and favoured the use of TENs [5].

Imam MA compares the outcome of spica cast versus TENS in pediatric femoral fractures in patients younger than 16 years and recommended use of TENS for fixation of such fractures [6].

Limb length discrepancy has been reported and is more often associated with femur fractures. Overlap at least 1.5 cm between fracture ends to prevent overgrowth has been advised. On end-to-end alignment, overgrowth is still a potential problem when using TEN [28-29]. These patients have to be followed up until they attain skeletal maturity. The implant should be removed after nine months of the postoperative period with a fully united fracture site or problems develop due to implants [30].

We have attained union in all our cases. Also, the functional outcome of the patients has been excellent in 25 patients (83.3%) and satisfactory in five patients (16.7%) as per Flynn criteria [7]. We did not face any complications that were out of proportion compared to published data regarding the use of TEN in pediatric long bone fractures. The complications, i.e., superficial infection, overgrowth, and prominent hardware causing irritation, were all dealt with timely and did not lead to any deterioration in the functional outcome of these children. Rehabilitation and patient care were less cumbersome for parents and complications related to cast care and cast loosening were avoided by the use of TEN for fracture stabilization.

Limitations

We accept the limitations of our study being retrospective, not having a control group, and not comparing with other methods of treatment. Also, our sample size was limited.

## Conclusions

We would suggest from our experience that TEN can become the first method of management of pediatric long bone fractures, especially in older children, more than six years and up to 16 years. Also, in cases where maintaining reduction with closed casting is a challenge (obese, open fracture, mentally retarded, or neuromuscular disorders) or hindrance to rehabilitation and nursing care, surgical management with TEN can be preferred. To prove better fracture stability and early return to function, we may need comparative studies with similar fractures managed with a plaster cast. However, the surgeon must be sound with technique as well as the limitations of these devices. Using an accurate technique and paying attention to the principles of fracture fixation methods with these elastic intramedullary nails, we can achieve good results and avoid common complications.

## Appendices

Table 1 provides the master datasheet.

**Table 1:** Master datasheet RTA: road traffic accident

Age	Sex	Bone	Mode of injury	Time to union (wk)	Limb length discrepancy	Rotational malalignment(degree)	Pain	Complication	
8	m	femur	Abuse	10	< 1 cm (1.2)	<5	present		
11	m	forearm	RTA	10	< 1 cm	<5	absent		
10	f	forearm	Self fall	8	< 1 cm	<5	absent		
10	m	femur	RTA	14	< 1 cm	<5	absent		
9	m	femur	RTA	8	< 1 cm	<5	absent	Hardware prominence	
12	m	tibia	RTA	8	< 1 cm	<5	absent		
8	m	femur	RTA	10	< 1 cm	<5	present	Superficial wound	
9	f	forearm	Self fall	8	< 1 cm	<5	absent		
9	f	femur	Fall from hieght	12	< 1 cm	<5	absent		
11	m	femur	RTA	16	< 1 cm	<5	absent	Hardware prominence	
8	m	femur	RTA	8	< 1 cm	<5	absent		
8	m	forearm	Fall from hieght	10	< 1 cm	<5	absent		
7	f	femur	RTA	6	< 1 cm	<5	absent		
7	m	femur	Fall from hieght	8		<5	absent		
9	m	femur	Fall from hieght	10	< 1 cm	<5	absent	Hardware prominence	
10	f	femur	RTA	10	< 1 cm	5 to 10	absent		
10	m	femur	RTA	12	< 2 cm (1.2)	<5	absent		
11	m	forearm	Self fall	10	< 1 cm	<5	absent		
14	f	tibia	RTA	12	< 1 cm	<5	present	Superficial wound	
8	m	femur	Fall from hieght	10	< 1 cm	<5	absent		
7	m	femur	Fall from hieght	10	< 1 cm	<5	present		
7	f	forearm	Self fall	6	< 1 cm	<5	absent		
13	m	tibia	RTA	12	< 1 cm	<5	present	Superficial wound	
6	m	femur	Fall from hieght	10	< 1 cm	<5	absent		
8	f	forearm	RTA	8	< 1 cm	<5	present	Hardware prominence	
7	m	forearm	Self fall	6	< 1 cm	<5	absent		
15	m	tibia	RTA	10	< 1 cm	<5	absent		
13	m	tibia	RTA	14	< 1 cm	<5	absent		
13	m	humerus	Abuse	12	< 1 cm	5 to 10	absent		
14	f	tibia	RTA	10	< 1 cm	<5	absent		
9.733333				9.933333					

## References

[1] McKibbin B (1978). The biology of fracture healing in long bones. J Bone Joint Surg Br.

[2] Saikia K, Bhuyan S, Bhattacharya T, Saikia S (2007). Titanium elastic nailing in femoral diaphyseal fractures of children in 6-16 years of age. Indian J Orthop.

[3] Nielsen AB, Simonsen O (1984). Displaced forearm fractures in children treated with AO plates. Injury.

[4] Sanders JO, Browne RH, Mooney F (2001). Treatment of femoral fractures in children by paediatric orthopaedists: results of 1998 POSNA survey. J Pediatr Orthop.

[5] Allen JD, Murr K, Albitar F, Jacobs C, Moghadamian ES, Muchow R (2018). Titanium elastic nailing has superior value to plate fixation of midshaft femur fractures in children 5 to 11 years. J Pediatr Orthop.

[6] Imam MA, Negida AS, Elgebaly A (2018). Titanium elastic nails versus spica cast in pediatric femoral shaft fractures: a systematic review and meta-analysis of 1012 patients. Arch Bone Jt Surg.

[7] Flynn JM, Hresko T, Reynolds RA, Blasier RD, Davidson R, Kasser J (2001). Titanium elastic nails for pediatric femur fractures, a multicenter study of early results with analysis of complications. J Pediatr Orthop.

[8] Rush LV (1951). Dynamic factors in medullary pinning of fractures. Am Surg.

[9] Ender J, Simon-Weidner R (1970). Die Fixenrung der tronchanteren bruche mit runden elastischen condylennageln [Article in German]. Acta Chir Austr.

[10] Ligier JN, Metaizeau JP, Prevot J, Lascombes P (1998). Elastic stable intramedullary nailing of femoral shaft fractures in children. J Bone Joint Surg Br.

[11] Saigal A, Agrawal AC (2013). Role of titanium elastic nailing in pediatric femoral shaft fractures. J Orthop Traumatol Rehabil.

[12] Ward WT, Levy J, Kaye A (1992). Compression plating for child and adolescent femur fractures. J Paediatr Orthop.

[13] Bar-On E, Sagiv S, Porat S (1997). External fixation or flexible intramedullary nailing of femoral shaft fractures in children. A prospective, randomized study. J Bone Joint Surg Br.

[14] Beaty JH, Austin SM, Warner WC, Canale ST, Nichols L (1994). Interlocking intramedullary nailing of femoral-shaft fractures in adolescents: preliminary results and complications. J Pediatr Orthop.

[15] Letts M, Jarvis J, Lawton L, Davidson D (2002). Complications of rigid intramedullary rodding of femoral shaft fractures in children. J Trauma.

[16] Buford D, Christensen K, Weather P (1998). Intramedullary nailing of femoral fractures in adolescents. Clin Orthop Relat Res.

[17] Flynn JM, Skaggs DL, Sponseller PD, Ganley TJ, Kay RM, Leitch KK (2002). The operative management of pediatric fractures of the lower extremity. J Bone Joint Surg Am.

[18] Narayanan UG, Hyman JE, Wainwright AM, Rang M, Alman BA (2004). Complications of elastic stable intramedullary nail fixation of pediatric femoral fractures and how to avoid them. J Pediatr Orthop.

[19] Lascombes P, Haumont T, Journeau P (2006). Use and abuse of flexible intramedullary nailing in children and adolescents. J Pediatr Orthop.

[20] Vrsansky P, Bourdelat MD (2000). Flexible stable intramedullary pinning technique in the treatment of pediatric fractures. J Pediatr Orthop.

[21] Vasilescu DE, Cosma D (2014). Elastic stable intramedullary nailing for fractures in children - principles, indications, surgical technique. Clujul Med.

[22] Sahu B, Mishra A, Tudu B (2018). Management of pediatric both-bone forearm fractures by titanium elastic nailing system: a prospective study of 40 cases. J Orthop Traumatol Rehabil.

[23] Kapila R, Sharma R, Chugh A, Goyal M (2016). Evaluation of clinical outcomes of management of paediatric bone forearm fractures using titanium elastic nailing system: a prospective study of 50 cases. J Clin Diagn Res.

[24] Zivanovic DV, Slavkovic AR, Radovanovic ZL, Marjanovic ZO, Bojovic NM, Djorjdevic IM, Petrovic MD (2018). Elastic stable intramedullary nailing of humerus fractures in children. Int J Clin Exp Med.

[25] Pogorelić Z, Kadić S, Milunović P, Pintarić I, Jukić M, Furlan D (2017). Flexible intramedullary nailing for treatment of proximal humeral and humeral shaft fractures in children: a retrospective series of 118 cases. Orthop Traumatol Surg Res.

[26] Goodwin RC, Gaynor T, Mahar A, Oka R, Lalonde F (2005). Intramedullary flexible nail fixation of unstable pediatric tibial diaphyseal fractures. J Pediatr Orthop.

[27] Sankar WN, Jones KJ, David Horn B, Wells L (2007). Titanium elastic nails for pediatric tibial shaft fractures. J Child Orthop.

[28] Corry IS, Nicol RO (1995). Limb length after fracture of the femoral shaft in children. J Pediatr Orthop.

[29] Macnicol MF (1997). Fracture of the femur in children. J Bone Joint Surg Br.

[30] Bhaskar A (2005). Treatment of long bone fractures in children by flexible titanium elastic nails. Indian J Orthop.

